# Risk Factors, Incidence, and Prognosis of Thromboembolism in Cancer Patients Treated With Immune Checkpoint Inhibitors

**DOI:** 10.3389/fphar.2021.747075

**Published:** 2021-11-08

**Authors:** Xue-lin Zou, Wei-yong Chen, Guang-yan Zhang, Hua Ke, Qiu-hong Yang, Xiao-bo Li

**Affiliations:** Department of Respiratory Medicine, Chengdu Seventh People’s Hospital, Chengdu, China

**Keywords:** immune checkpoint inhibitors (ICIs), incidence, risk factors, thromboembolism, prognosis

## Abstract

In recent years, immune checkpoint inhibitors (ICIs) have become the standard treatment option for tumors. With the widespread application of ICIs, immune-related adverse events (irAEs) have gradually attracted the attention of researchers. Owing to the characteristics of ICIs, irAEs can affect each organ of the human body. Thromboembolism is uncommon in cancer patients receiving ICIs, but it may affect their survival. Most thromboembolic events do not cause serious effects after early prediction and treatment, but life-threatening toxic reactions are also observed. This condition should not be ignored because of vague and atypical symptoms, which make early diagnosis more challenging. This article focuses on the high-risk factors, underlying mechanisms, incidence, and prognosis of thromboembolism in patients using ICIs and briefly describes the intervention and treatment measures. This information would allow patients to effectively manage the side effects of thromboembolism during Immune checkpoint inhibitors treatment, ensuring the efficacy of ICIs and reducing mortality.

## 1 Introduction

Immune checkpoint inhibitors (ICIs) are widely used in tumor treatment. They inhibit the negative costimulation of T cells, help reactivate T cells, enhance their anti-tumor effects, and improve the survival of patients (Postow et al., 2015). There are several types of ICIs. The commonly used ICIs target the programmed cell death receptor-1 (PD-1), programmed cell death ligand-1 (PD-L1), and cytotoxic T lymphocyte associated antigen 4 (CTLA-4) (Postow et al., 2015; Hoos, 2016).

With the widespread use of ICIs, adverse reactions in organs have attracted the attention of doctors and researchers. One of the most important complications in cancer is thromboembolism (TE) (Postow et al., 2018). A series of studies have explored the frequency and mechanism of venous TE (VTE) in patients receiving ICIs. Research has revealed that the incidence of VTE is as high as 30.3% (Roopkumar et al., 2018), which indicates that ICIs may affect the coagulation pathway (Roopkumar et al., 2021). A possible mechanism is where activated T cells promote procoagulant activity by inducing coagulation-initiating tissue factor (TF) in antigen-presenting cells, which indicates that there may be a correlation between ICIs and TE events (Del Prete et al., 1995; Iqbal et al., 2014; Jiang et al., 2014).

TE includes VTE and arterial TE (ATE). VTE includes acute symptomatic or occasional deep vein thrombosis, pulmonary embolism (PE), visceral vein thrombosis, and fatal PE (Ay et al., 2010). ATE is defined as acute coronary syndrome, acute peripheral arterial occlusion, and ischemic stroke (Grilz et al., 2018). The possible symptoms of TE include dyspnea and swelling of the lower limbs, which are not obvious and specific (Guven et al., 2021). In addition, most VTE cases are detected incidentally, and they may be ignored in the clinic or in research (Guven et al., 2021). What needs more attention is that one study has reported fatal TE events during ICI therapy (Moik et al., 2021). However, it is not clear which high-risk factors for TE exist for patients receiving ICIs, and the impact on the prognosis is still controversial. In this review, we introduce the pathogenesis of TE upon ICI treatment in detail, as well as potential risk factors, morbidity, impact on prognosis, and corresponding preventive measures.

## 2 Mechanism of TE

**FIGURE 1:**
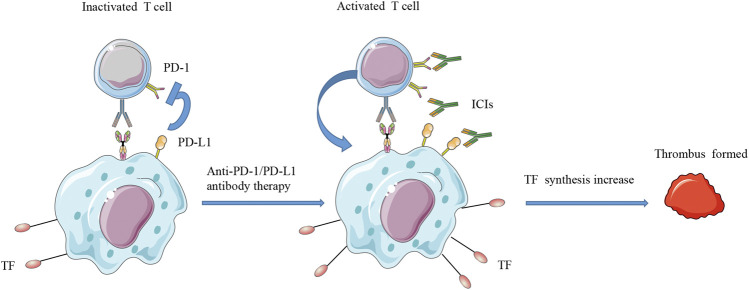
Schematic diagram of T cell activation and increased TF synthesis. PD-L1 can bind to tumor-specific T cells to inhibit their immune function. When ICIs remove this inhibitory state, T cell activation will induce the expression of TF on PD-L1-positive monocytes. Therefore, T cells activated by ICIs may induce a large amount of TF production in monocytes and may lead to dysfunction of the coagulation system. ICIs, immune checkpoint inhibitors; PD-L1, programmed cell death ligand 1; PD-1, programmed death receptor 1; TF, tissue factor.

The coagulation-fibrinolysis process maintains a balanced state of normal physiological functions, but its function changes in malignant tumors (Varki, 2007; Repetto and De Re, 2017). The mechanism by which ICI affects the immune system and causes thrombosis is not fully understood (Postow et al., 2018). One possible mechanism is that activated T cells induce increased production of TF to promote procoagulant activity (Varki, 2007; Teraoka et al., 2017; Haratani et al., 2018; Sato et al., 2019b). TF is mainly derived from monocytes (Gregory et al., 1989; Varki, 2007; Kasthuri et al., 2009). It is a transmembrane cell surface glycoprotein that can trigger an exogenous coagulation cascade to achieve hemostasis (Mackman, 2004; Kasthuri et al., 2009). PD-L1 binds to tumor-specific T cells to inhibit immune function (Kuang et al., 2009; Francisco et al., 2010). When ICIs remove this inhibitory state, T cell activation induces the expression of TF in PD-L1-positive monocytes. In particular, PD-L1-high monocytes express more TF than PD-L-low monocytes (Sato et al., 2019a). Therefore, T cells activated by ICIs may induce a large amount of TF production in monocytes and lead to dysfunction of the coagulation process (Figure 1).

The biomarkers observed in another study may provide a possible mechanism for the occurrence of VTE in patients receiving ICIs (Roopkumar et al., 2021). Myeloid-derived suppressor cells (MDSCs) are derived from the bone marrow and are the precursors of dendritic cells, macrophages, and granulocytes (Gabrilovich, 2017). They are associated with tumor progression and metastasis (Ruffell and Coussens, 2015; Munn and Bronte, 2016; Gabrilovich, 2017; Tobin et al., 2018) and are key targets of immunotherapy (Liu et al., 2018; Loeuillard et al., 2020). These cells regulate their procoagulant activity and may impair anti-tumor immunity (Graf et al., 2019). IL-8 released by tumors and other cells in the tumor microenvironment is closely related to the accumulation of MDSCs in the tumor (Alfaro et al., 2016). Furthermore, expressions of IL-8 and many other inflammatory cytokines are elevated in patients with TE receiving immunotherapy, which may directly act on endothelial cells and other cells to induce pro-inflammatory and procoagulant reactions (Boulanger, 2016; Sturtzel, 2017). IL-8 can also activate CXC chemokine receptor 1/MDSCs and induce tumor infiltration to release neutrophil extracellular traps, which play a key role in thrombotic inflammatory diseases (Ekdahl et al., 2016; Foley and Conway, 2016). In addition, MDSCs can promote platelet aggregation (Boutté et al., 2011) and IL-8 can induce platelet activation and spread (Bester and Pretorius, 2016).

The above studies revealed a possible mechanism of VTE in tumor patients treated with ICIs. The relationship between ICI and atherosclerosis is well-established (Foks and Kuiper, 2017); therefore, we have not elaborated on the mechanism of ATE. Further preclinical research is warranted to reveal the mechanism of TE in cancer patients receiving ICI therapy.

## 3 Risk Factors for TE

TE in cancer patients receiving ICI therapy may be caused by different high-risk factors, but this has not been fully studied. The following sections summarize some common high-risk factors based on published research.

### 3.1 Previous Comorbidities of Patients

Previous studies have shown that a history of VTE or ATE is a risk factor in cancer patients receiving ICI therapy (Bar et al., 2019; Ando et al., 2020; Gutierrez-Sainz et al., 2021; Guven et al., 2021; Moik et al., 2021). Another study showed that a history of coronary artery disease is also significantly associated with VTE (Sussman et al., 2021). A retrospective study indicated that a history of heart disease may be a risk factor for thromboembolic events (Ando et al., 2020). However, according to the results published by Nichetti et al., the previous occurrence of TE events is not a high-risk factor for newly diagnosed thromboembolic events (Nichetti et al., 2019). Based on the conclusions of most of the above studies, patients with a history of TE events or heart disease should frequently undergo computed tomography examination or take preventive anticoagulants.

### 3.2 Tumor Stage

The presence of a stage IV tumor is also one of the reasons for the increased risk of TE in cancer patients receiving ICI treatment. In a retrospective cohort study, patients with stage IV tumors had an increased risk of TE (Kewan et al., 2021). Similar results have been reported in the literature indicating that stage IV and/or metastatic disease are closely related to VTE (Moik et al., 2021; Roopkumar et al., 2021). According to a retrospective study, although the incidences of VTE in patients with adenocarcinoma and squamous cell carcinoma are 6.7 and 2.1%, respectively, the incidence of VTE in patients with distant metastasis is approximately 22.0% (Blom et al., 2004). Another retrospective study also showed that the incidence of VTE in patients with stage IV melanoma was 25.2% (Sparsa et al., 2011). In contrast, the risk factors for more than two cancer metastases are related to the risk of a reduction in TE (Nichetti et al., 2019). Overall, stage IV and/or metastatic disease are high-risk factors for TE.

### 3.3 Khorana Score (KS)

KS, a risk stratification tool, has been used for VTE risk assessment (Traill and Gleeson, 1998; Key et al., 2020). It includes clinical parameters (cancer type, body mass index) and laboratory parameters (hemoglobin, white blood cell, and platelet count). According to the score, patients are assigned into low-, medium-, and high-risk groups. However, almost all current relevant studies have found that KS is not efficient for identifying cancer patients suitable for VTE prevention (Nichetti et al., 2019; Icht et al., 2021; Kewan et al., 2021; Moik et al., 2021). Only one study has shown that a KS ≥ 1 is significantly associated with VTE (Sussman et al., 2021). Most studies did not detect a good predictive power of KS because the risk assessment model was developed for patients undergoing chemotherapy (Khorana et al., 2008; Verso et al., 2012). Therefore, it is necessary to develop ICI-specific VTE risk assessment models to assist in clinical treatment decisions.

### 3.4 Eastern Cooperative Oncology Group (ECOG) Performance Status (PS)

The ECOG status is closely related to the treatment and survival of patients (Scotté et al., 2019). Poor ECOG status is associated with an increased risk of VTE, which has been previously confirmed in patients receiving chemotherapy (Ferroni et al., 2015). However, few studies have incorporated baseline ECOG fitness status into the analysis of risk factors for TE formation, and the conclusion are inconsistent. Guven et al. found that the risk of venous thrombosis is significantly increased when the patient’s baseline ECOG PS is one or more (*p* = 0.048) (Guven et al., 2021). In contrast, the results reported by Moik et al. proved that patients with different ECOG statuses have similar risks of VTE (0 and ≥1, *p* = 0.760) (Moik et al., 2021). Patients with ECOG PS ≥ 2 did not have any VTE events, which may be due to the shorter follow-up period (Nichetti et al., 2019).

### 3.5 ICI Combination Therapy

A previous study showed that combined ICI therapy is significantly associated with VTE (Sussman et al., 2021). Two patients with glioblastoma multiforme, who had been treated with bevacizumab before immunotherapy, developed VTE (Guven et al., 2021). The combination of anti-VEGF therapies and ICIs needs to be investigated more as it was shown previously that for patients that received pembrolizumab combined with axitinib, VTE was unreported (Rini et al., 2019). ICI combination therapy may be responsible for the increased incidence of TE. However, data from several retrospective analyses indicated that treatments combining ICIs with targeted therapies or chemotherapy are not related to the high risk of VTE or ATE (Roopkumar et al., 2018; Nichetti et al., 2019; Guven et al., 2021). Compared with that in ICI monotherapy, the incidence of ATE in combination therapy is higher, although not statistically significant (Sussman et al., 2021). It is important to consider that combination with ICI therapy may increase the risk of TE.

### 3.6 Different Types of Tumors

Associations between the type of cancers and the risk of thrombosis have been compared. The prediction score includes the location of the primary tumor as a variable for predicting TE (Khorana et al., 2008). Lung cancer and melanoma account for a large proportion (Ibrahimi et al., 2017; Roopkumar et al., 2018; Sussman et al., 2020). Bar et al. assessed the risk of VTE and found that lung adenocarcinoma is significantly associated with the occurrence of VTE (Bar et al., 2019). Patients with gynecological cancer or melanoma have a higher risk of developing VTE than other patients (Gutierrez-Sainz et al., 2021; Moik et al., 2021). However, in another study, the incidence of VTE was reported to be similar between different cancer types (Kewan et al., 2021). At present, few studies are focusing on this area, and more prospective or randomized controlled studies are required to confirm this.

### 3.7 Other High-Risk Factors

To date, evidence supporting that certain types of ICIs cause TE in patients is insufficient (Kewan et al., 2021; Moik et al., 2021; Roopkumar et al., 2021). Sex, specifically for females, was identified as the covariate with a significant association with TE (Roopkumar et al., 2018; Gutierrez-Sainz et al., 2021). In addition, one study has shown that smoking status and a high PD-L1 expression (>50%) increase the risk of developing VTE (Nichetti et al., 2019). Hypertension and dyslipidemia are also associated with VTE (Bar et al., 2019). Age is also included in the analysis as a high-risk factor (*p* < 0.05) (Roopkumar et al., 2021). So far, the data published on TE events related to ICI are sparse and conflicting, because current studies have not paid attention to providing this type of data for clinical reference. In future research, it is necessary to list the above risk factors or more data on each risk factor, and further determine the final risk factor, as well as provide a basis for the use of anticoagulation therapy for high-risk groups.

## 4 Incidence of TE in Patients Treated With ICIs and Its Impact on the Prognosis

ICI therapy has demonstrated profound clinical efficacy for multiple malignancies, which may also introduce additional risks. An increasing number of issues are of great interest in the incidence of TE and their impact on the prognosis of cancer patients treated with ICIs. Data from the retrospective studies summarized in this review suggest that the incidence of VTE is between 2.6 and 30.3% (Supplementary Table S1). It has been recognized that cancer patients also have an increased risk of ATE, with an incidence of 1.2–6.1% (Ando et al., 2020; Kewan et al., 2021; Moik et al., 2021; Sussman et al., 2021). In addition, case reports describing the occurrence of ATE related to the use of ICIs provide further speculation about the potential increase in the underlying ATE risk (Boutros et al., 2018; Roopkumar et al., 2018; Roopkumar et al., 2021).

We summarized the incidence of TE events in cancer patients receiving ICI therapy from published articles (Supplementary Table S1). Evidence on whether TE has a negative impact on survival varies. According to the data summarized in Supplementary Table S1, lung cancer is the most prevalent type, which was incorporated into several retrospective studies. A retrospective cohort study included patients with lung cancer treated with nivolumab and provided the cumulative incidence (CI) of TE (18.4%) with a median follow-up of 10.8 months after starting nivolumab (Hegde et al., 2017). According to a study involving 345 patients with non-small cell lung cancer (NSCLC), the 6-months CI of VTE in the ICI cohort was 4.5%, and a strong association between VTE and death was confirmed (Icht et al., 2021). A recent study of patients with NSCLC receiving immunotherapy showed that the incidence of VTE was 13.8% (Nichetti et al., 2019). Sato et al. found that 12% of patients with NSCLC receiving ICI monotherapy were diagnosed with coagulation-fibrinolysis system-related diseases (Sato et al., 2019b). Another retrospective study involving 228 patients with melanoma reported that the incidence of TE events was 20.6%, including 16.2% VTE and 6.1% ATE events (Sussman et al., 2021). In patients without brain metastases, the incidence of TE is significantly associated with a decrease in overall survival (OS) (Sussman et al., 2021).

According to the rest of the data from the remaining studies, which included various kinds of cancers, lung cancer is the most frequent tumor treated with ICIs, followed by melanoma and renal cell carcinoma. Bar et al. evaluated the incidence of TE after ICI administration in 2.6% of patients, and the event rate was 5.2% in the lung adenocarcinoma cohort (Bar et al., 2019). The survival of patients with TEs was worse than that of those without TE (Bar et al., 2019). Gutierrez-Sainz et al. found that 16 of 229 patients (7%) developed VTE, which was not independently associated with shorter OS (*p* = 0.44) (Gutierrez-Sainz et al., 2021). The information provided by Ando et al. showed that cancer-associated thrombosis incidence was 8.2% in patients administered nivolumab or pembrolizumab (Ando et al., 2020). Ibrahimi et al. concluded that the incidence of VTE was 10% (16/154 cases), which was not significantly correlated with progression-free survival (*p* = 0.725) (Ibrahimi et al., 2017). Another retrospective study showed that the incidence of VTE in cancer patients who received ICI treatment was 10.5% (Kewan et al., 2021). However, the median OS of patients with and without VTE within 3 months was not statistically significant (Kewan et al., 2021). A retrospective study conducted by Guven et al. showed that the most frequent tumors are renal cell carcinoma (26.3%) and melanoma (24.1%), and that the incidence of VTE is 11.3% (Guven et al., 2021). The median survival of patients with VTE was shorter than that of those without VTE, but the difference was not statistically significant (Guven et al., 2021). Moik et al. enrolled 672 patients, and the results showed that the CI of VTE was 12.9% (Moik et al., 2021). The incidence of VTE was associated with increased mortality (Moik et al., 2021). The incidence of VTE was 24 and 30.3%, respectively, in two other studies, which were significantly higher than that reported in previous studies (Roopkumar et al., 2018; Roopkumar et al., 2021). After comparing with other studies, we found several common points. First, the proportion of patients with metastasis is high (88–90.3%). Second, nivolumab is the most commonly used single-drug immunotherapy (51.5–52.3%). Third, the lung is the most common primary site of cancer (49.6%). However, a high occurrence of VTE is inconsistent with OS (Roopkumar et al., 2018). In another study, the incidence of VTE was 24%, which was associated with a decrease in OS (*p* < 0.008) (Roopkumar et al., 2021).

The data available for ATE are limited. In one study, only six (1.1%) and one (0.18%) patient had ischemic stroke and arterial thrombosis, respectively (Kewan et al., 2021). Similarly, another retrospective research demonstrated that the incidence of ATE was 1.8% (Moik et al., 2021). Ando et al. evaluated 122 eligible participants treated with ICIs and showed that the incidence of ATE was 4% (Hu et al., 2017). Likewise, the incidence of ATE in another retrospective study was 6.1% (Sussman et al., 2021). The result of an epidemiological study showed that the incidence of ATE in cancer patients newly diagnosised within 6 months was more than twice that of non-cancer patients in the control group. Moreover, the risk of death of ATE in cancer patients is tripled (Navi et al., 2017).

## 5 Prevention and Anticoagulation Treatment

In cancer patients treated with ICIs, it is not clear whether the use of anticoagulation therapy can effectively reduce the incidence of TE. Previous studies have shown that through VTE preventive anticoagulation therapy, the incidence of VTE in high-risk cancer patients undergoing conventional chemotherapy has been reduced by 6% (Carrier et al., 2019; Khorana et al., 2019). In a retrospective study, the incidence of TE (20.6%) in melanoma patients during ICI treatment was higher than that in those undergoing conventional chemotherapy (Sussman et al., 2021). In another study, the incidence of ATE after ICI administration was 4.9% (Ando et al., 2020). This is higher than the incidence of ATE after Bevacizumab treatment (3.8%) (Scappaticci et al., 2007). Recent studies have shown that preventive anticoagulation therapy can prevent VTE in cancer patients (Carrier et al., 2018; Khorana et al., 2019; Roopkumar et al., 2021). Therefore, thrombosis prevention may be considered for the patients receiving ICIs. In some studies, anticoagulation therapy was mainly used in patients with a history of VTE at the beginning of ICI treatment, and it was found that the incidence of VTE increased instead. The possible reason that cannot be ignored is that not all patients continue to use anticoagulant therapy during ICI treatment (Ando et al., 2020; Kewan et al., 2021; Sussman et al., 2021). Data from a retrospective analysis showed that patients with a history of VTE were administered preventive anticoagulants, and the use of preventive anticoagulants was not associated with the risk of VTE (*p* = 0.675) (Guven et al., 2021). Therefore, these findings indicate that anticoagulation therapy is mainly used in patients with a history of TE (Guven et al., 2021). In addition, attention should also be paid to the occurrence of ATE when using ICI, as is the case with VEGF inhibitors, and it is necessary to fully evaluate whether patients need preventive anticoagulation therapy.

A retrospective study aimed to assess the risk of intracranial hemorrhage in patients with melanoma brain metastasis after anticoagulation therapy. Among 57 patients (77%) who received anticoagulation therapy, two (4%) developed intracranial hemorrhage. There was no significant difference between these patients and those who did not receive anticoagulation therapy. In addition, VTE patients who received anticoagulant therapy achieved longer OS (Alvarado et al., 2012). However, the safety of anticoagulation therapy still needs to be fully evaluated in tumor patients receiving ICI therapy. In one study, it was shown that patients with VTE were more likely to experience clinically significant bleeding during treatment than patients in the non-VTE cohort (41.4 vs. 18.4%, *p* = 0.00004) (Kewan et al., 2021). The risk of bleeding may be because of anticoagulant therapy, rather than VTE. Another retrospective study showed that among 47 patients with VTE, 8.5% showed VTE recurrence and 12.8% had bleeding during anticoagulation (Sato et al., 2019b). In view of the high risk of re-thrombosis and severe bleeding after anticoagulation therapy, managing TE is a challenging task (Prandoni et al., 2002). Therefore, patients receiving ICI therapy need to be evaluated in advance and should be cautiously administered preventive anticoagulation therapy.

## 6 Conclusion

According to the data summarized in this study, TE incidence is relatively high incidence in cancer patients treated with ICI, even exceeding that in patients receiving chemotherapy (Sussman et al., 2021). This review identified an important issue in a complex patient population, reminding clinicians to pay more attention to these cases, for which early detection and intervention should be implemented.

The included studies have some limitations. They included patients with tumor heterogeneity, such as inconsistent tumor types. In addition, the retrospective nature of these studies and the short follow-up time may have led to the loss of key data. Moreover, the ATE events are not sufficient to perform additional data analysis on susceptibility factors and survival analysis of specific tumor types. Therefore, future studies with larger sample sizes and prospective designs are essential to better describe TE in patients treated with ICIs. Preclinical studies should focus on the identification of risk factors and biomarkers for developing TE risk assessment models and screening patient groups at risk. The specific mechanism of bleeding during ICI treatment needs further elucidation. A unified thrombosis risk assessment standard for patients treated with ICIs should be established, for grading the risk level and to help obtain reliable and comparable data in the clinic. These would ensure that the risk of thromboembolism is avoided as much as possible, the therapeutic effect of ICIs is maximized, and the OS is prolonged.
