# Supplementary material for: Risk Factors, Incidence, and Prognosis of Thromboembolism in Cancer Patients Treated With Immune Checkpoint Inhibitors
Source: Front Pharmacol. 2021 Nov 8;12:747075. doi: 10.3389/fphar.2021.747075 (PMC8606686; doi:10.3389/fphar.2021.747075)
Supplement: Supplementary file 1 [file Table1.docx]

**Supplementary Table S1** Summary of characteristics of the retrospective cohort studies cited in the text on the incidence of TE in cancer patients receiving ICIs treatment.

| Reference | Time | Cancer type | Stage | Sample size | Median age (year) | Male (%) | Median follow-up time (months) | TE, n (%) | High–risk factor | OS hazard ratio (HR) (95% CI) for TE |
| --- | --- | --- | --- | --- | --- | --- | --- | --- | --- | --- |
| Sami et al. | 2017 | Various | NR | 154 | 63 (23–89) | 43% | 7 | VTE 16 (10%) | NR | 150 days vs.146 days (p=0.725) |
| Hegde et al. | 2017 | Lung cancer | NR | 76 | NR | NR | 10.8 | TE 18.4% | Female | NR |
| Roopkumar et al. | 2018 | Various | Metastatic (88.7%) | 522 | 64 (10–91 ) | 58.8% | NR | VTE 30.3% | NR | 1.215 ( 95% CI, 0.94 –1.55, p = 0.121) |
| Sato Ryo et al. | 2019 | NSCLC | Advanced NSCLC | 83 | 70.5 (48–81) | NR | NR | VTE 10 (12%) | High PD-L1 expression | NR |
| Bar et al. | 2019 | Various | IV | 1215 | 65 | 59.0% | NR | VTE 2.60% | Lung adenocarcinoma, prior TE, hypertension and dyslipidemia | 3.01 (95% CI, 2.07–4.39, p <0.0001) |
| Ando et al. | 2019 | Various | NR | 122 | 75 | 74.6% | NR | TE 8.2% (VTE 4.1%, ATE 4.9%) | History of TE | NR |
| Nichetti et al. | 2019 | NSCLC | Metastatic (95.4 %) | 217 | 70 (32–90) | 62.7% | 37.8 (22.6–43.9) | TE 13.80% | Current smoking status, high (>50%) PD-L1 | 0.59 ( 95% CI, 0.37–0.93, p = 0.02 ) |
| Sussman et al. | 2020 | Melanoma | IV | 228 | 65 (23–91) | 67.5% | 27.3 | TE 20.6% (VTE 16.2%, ATE 6.1%) | Combination ICI, Khorana Score ≥1, history of coronary artery disease, anticoagulation at treatment start | 2.27 (95% CI, 1.36 - 3.79, p=0.002) * |
| Gutierrez-Sainz et al. | 2020 | Various | IV (96.5%) | 229 | 64 (19–86 ) | 63.8% | 9.8 | VTE 16 (7%) | Female, melanoma | 1.33 (95% CI, 0.63–2.80, p = 0.44) |
| Moik et al. | 2021 | Various | IV (86 %) | 672 | 64 (54–72) | 61.30% | 8.5 | VTE 47 (cumulative incidence 12.9%), ATE 9 (cumulative incidence1.8%) | None | 3.09 (95%CI, 2.07-4.60) |
| Icht et al. | 2021 | NSCLC | IV (85.8%) | 176 | 66 | 62.2% | 187 | VTE cumulative incidence (6 months) 4.5% | None | 12.2 (95% CI, 4.58-32.43) |
| Kewan et al. | 2021 | Various | IV | 552 | 68.8 (26.8–94.8) | 65.0% | 12.1 | VTE 58 (10.5%), ATE 7 (1.2%) | Anticoagulation use at the time of ICI treatment start | 13.4 months vs.12.8 months (p =0.791) |
| Guven et al. | 2021 | Various | IV | 133 | 60 (48–66) | 64.7% | 10.1 (5.8-18.5) | VTE 15 (11.3%) | ECOG ≥1 | 1.208 ( 95% CI, 0.577–2.526, p=0.616) |
| Roopkumar et al. | 2021 | Various | IV (90.3%) | 1686 | 64.5 (18–93) | 60.1% | 438 days (7–1,971 days) | VTE 404 (24%) | Age, the presence of advanced tumor stage and/or metastatic disease | 1.22 (95% CI, 1.06–1.41,p<0.008) |
|  |  |  |  |  |  |  |  |  |  |  |

ATE, arterial thrombosis; CI, confidence interval; ECOG, Eastern Cooperative Oncology Group; ICIs, immune checkpoint inhibitors; NSCLC, non–small cell lung cancer; NR, not report; OS, overall survival; TE, thromboembolism; VTE, venous thromboembolism.
